# Combination of peptide receptor radionuclide therapy with fractionated external beam radiotherapy for treatment of advanced symptomatic meningioma

**DOI:** 10.1186/1748-717X-7-99

**Published:** 2012-06-21

**Authors:** Michael C Kreissl, Heribert Hänscheid, Mario Löhr, Frederik A Verburg, Markus Schiller, Michael Lassmann, Christoph Reiners, Samuel S Samnick, Andreas K Buck, Michael Flentje, Reinhart A Sweeney

**Affiliations:** 1 Department of Nuclear Medicine, University Hospital Wuerzburg, Wuerzburg, Germany; 2 Department of Radiation Oncology, University Hospital Wuerzburg University of Wuerzburg, Josef-Schneider-Str. 11 97080, Wuerzburg, Germany; 3 Department of Neurosurgery, University Hospital Wuerzburg, Wuerzburg, Germany; 4 Department of Nuclear Medicine, RWTH University Hospital Aachen, Aachen, Germany

**Keywords:** PRRT, Peptide receptor radionuclide therapy, Meningioma, Radiotherapy, EBRT, Combination

## Abstract

**Background:**

External beam radiotherapy (EBRT) is the treatment of choice for irresectable meningioma. Due to the strong expression of somatostatin receptors, peptide receptor radionuclide therapy (PRRT) has been used in advanced cases. We assessed the feasibility and tolerability of a combination of both treatment modalities in advanced symptomatic meningioma.

**Methods:**

10 patients with irresectable meningioma were treated with PRRT (^177^Lu-DOTA0,Tyr3 octreotate or - DOTA0,Tyr3 octreotide) followed by external beam radiotherapy (EBRT). EBRT performed after PRRT was continued over 5–6 weeks in IMRT technique (median dose: 53.0 Gy). All patients were assessed morphologically and by positron emission tomography (PET) before therapy and were restaged after 3–6 months. Side effects were evaluated according to CTCAE 4.0.

**Results:**

Median tumor dose achieved by PRRT was 7.2 Gy. During PRRT and EBRT, no side effects > CTCAE grade 2 were noted. All patients reported stabilization or improvement of tumor-associated symptoms, no morphologic tumor progression was observed in MR-imaging (median follow-up: 13.4 months). The median pre-therapeutic SUV_max_ in the meningiomas was 14.2 (range: 4.3–68.7). All patients with a second PET after combined PRRT + EBRT showed an increase in SUV_max_ (median: 37%; range: 15%–46%) to a median value of 23.7 (range: 8.0–119.0; 7 patients) while PET-estimated volume generally decreased to 81 ± 21% of the initial volume.

**Conclusions:**

The combination of PRRT and EBRT is feasible and well tolerated. This approach represents an attractive strategy for the treatment of recurring or progressive symptomatic meningioma, which should be further evaluated.

## Introduction

While surgery is the mainstay in treatment of meningioma, external beam radiotherapy (EBRT) offers the only other curative option in meningioma management [1,2].

For benign (WHO Grade 1) tumors, recommended doses are generally 50–54 Gy in fractions of 1.8–2 Gy, alternatively single doses of 12–16 Gy are used in radiosurgery [1-3]. No clear dose–response relationship has been established justifying higher doses to date.

However, especially higher grade meningiomas (≥WHO II), large, irregular tumors in critical locations and recurrent tumors in both radiation-naive as well as previously irradiated regions would likely benefit from a highly conformal dose escalation without increasing dose to normal tissues [4,5]

In case of a recurrence after external radiotherapy, results of studies using pharmacological targeted approaches have been modest at best and are often associated with significant toxicities [6].

A potential target for a specific and well tolerable therapy in advanced meningioma is the somatostatin receptor (SSR). About 90% of the meningiomas show SSR expression, especially the receptor subtypes 1 and 2 [7]. Positron emission tomography using ^68^ Ga-labelled somatostatin analogues (SSR-PET) and single photon emission computed tomography (SPECT) using ^111^In-octreotid are well established for imaging neuroendocrine tumors and have also shown very promising results for the diagnostic work up and for target volume delineation in radiotherapy treatment planning of meningioma [8-10]. Since many tumors display a fairly strong expression of somatostatin receptors, “peptide receptor radionuclide therapy” (PRRT), which is well evaluated and commonly used in neuroendocrine tumors [11,12], has already been used in advanced cases of meningioma showing promising results [13-15]. However, in the largest study, published by Bartolomei et al., about one third of the patients showed tumor progression after therapy [15].

In order to increase efficacy of treatment, it seems reasonable to combine external beam radiotherapy and PRRT therapy. Besides delivering a higher radiation dose to the tumor, this approach could also be used to reduce the external beam radiation dose to critical organs at risk next to the tumor such as cranial nerves, brainstem and normal brain. Due to the short range of beta particles, somatostatin analogues labelled with [^177^Lu], such as ^177^Lu- DOTATATE (^177^Lu DOTA0,Tyr3 octreotate) and [^177^Lu]-DOTATOC (^177^Lu-DOTA0,Tyr3octreotide) are of special interest.

We herein report the tolerance and feasibility of a combined PRRT-EBRT approach with one cycle of radiopeptide therapy preceeding EBRT in patients with symptomatic non-resectable meningioma.

## Materials and methods

### Patients

From May 2010 to May 2011, 10 patients with irresectable advanced primary or recurrent meningioma (7 WHO grade I, 2 grade II, 1 not known) were treated with radiopeptide therapy and EBRT. As part of treatment planning, all patients received somatostatin receptor PET to assess not only SSR expression but also for a more accurate delineation of the target volume. All cases were initially discussed and assigned to the pilot trial in unanimous decision in an interdisciplinary tumor board including physicians from neurosurgery, radiation oncology, neuroradiology, nuclear medicine and medical oncology. The combined treatment was performed according to German law on a compassionate use basis. All patients gave their informed consent for the procedure. Patient characteristics can be found in Table 1. All except one patient had one or more previous surgeries; one had been previously treated with radiotherapy.

**Table 1:** Patient baseline characteristics

**Pat. no.**	**Age (y)**	**Sex**	**WHO grade**	**Karnofsky score**	**Meningioma location**	**Prior Resection**	**Prior RT**	**Mag3 Clearance (% of norm)**	**PTV (ml)**	**Indication for intensified protocol / clinical presentation**
1	27	f	II	90	Right sphenoid wing	2	-	67.7	115	Large tumor, grade II, age/ pituitary insufficiency, decreased vision /ptosis
2	55	f	I	100	Both sphenoid wings	1	0	82.4	223	Very large tumor / retroorbital pressure,
3	68	f	NA*	90	Left petroclival	0	0	73.9	73	Critical location / Paresis of CN III, VI & VII
4	53	f	I	80	Right sphenoid wing	2	0	87.1	38	Critical location / exophthalmus and chemosis right eye, progressive loss of vision
5	52	f	I	100	Both sphenoid wings	1	0	61.7	197	Large tumor critical location /, progressive loss of vision
6	65	f	I	100	Right sphenoid wing	1	0	83.2	29.3	Critical location /trigeminal hypesthesia
7	62	f	I	80	Left falx & vertexregion	1	0	89.4	139	Clear-cell component, quickly progressive hemiparesis
8	63	m	II	90	Falx central region	1	1	112.7	28	Previous RT, Grade II, large tumor / paresis left leg
9	69	f	I	90	Left olfactory nerve & neurinoma of ethmoidal sinus^§^	1	0	82.9	121	Large tumor/ epilepsy, tremor
10	67	m	I	90	Multifocal bifrontal to right sphenoid wing	2	0	108.3	294	Ver large tumor/ dizziness, swelling and ptosis right palpebra

* Biopsy not possible.

^§^ SSR-expression only in the meningioma, radiotherapy of both, meningioma and neurinoma.

^#^ Ga-DOTATOC.

^+^ Ga-DOTATATE.

CN = Cranial Nerve.

### PET imaging and PRRT

All patients were assessed for somatostatin receptor expression by PET prior to initiation of PRRT. Until September 2010 [^68^ Ga-DOTA0,Tyr3]octreotide (Ga-DOTATOC) was employed; due to a change in availability of the precursor peptide, [^68^ Ga-DOTA0,Tyr3]octreotate (Ga-DOTATATE) was used thereafter. The somatostatin analogue for PRRT was switched simultaneously for the same reason.

Also, until September 2010, a dedicated PET (Siemens ECAT Exact 47) was used for SSR-PET in the first 6 patients; afterwards a newly installed PET/CT (Siemens Biograph mCT 64) was used.

Data sets of the head were acquired 60 minutes after the injection of 111 ± 35 MBq ^68^ Ga-DOTATOC/DOTATATE. For the dedicated PET, 3D data were collected for 20 min and corrected for attenuation using Ge-68 line sources, data were reconstructed using filtered back projection.

On the PET/CT, a 10 min 3D data acquisition (matrix: 200 x 200) was started after a low-dose-CT (30 mAs, 120 kV, slice thickness 0.5 mm). After decay and scatter correction, PET data were reconstructed iteratively with attenuation correction using dedicated software (HD.PET, Siemens Esoft).

The maximum standardized uptake value SUV_max_ as well as the mean SUV in a spherical volume of interest 1 cm in diameter (SUV_peak_) in the tumor region with highest tracer uptake were determined (Table 2).

**Table 2:** Details on PET diagnostics

		**PET before therapy**				**Follow-up PET**				
Patient no.	Scanner	^68^ Ga- peptide	SUV_max_	SUV_peak_	Volume ³	Scanner	^68^ Ga- peptide	SUV_max_	SUV_peak_	Volume ³
1	ECAT	DOTATOC	11.4	10.0	28.9	ECAT	DOTATOC	18.0	14.9	25.2
2	ECAT	DOTATOC	44.8	38.8	11.8	ECAT	DOTATATE	54.1	47.9	12.6
3	ECAT	DOTATOC	13.1	11.4	9.6	mCT	DOTATATE	20.3	15.3	7.9
4	ECAT	DOTATOC	4.3	3.5	7.7	mCT	DOTATATE	8.0	5.3	3.5
5	mCT	DOTATOC	34.2	29.8	32.6	mCT	DOTATATE	40.4	32.1	34.2
6	ECAT	DOTATOC	68.7	59.7	4.1	mCT	DOTATATE	119.0	87.3	3
7	mCT	DOTATATE	14.6	13.1	7.1	mCT	DOTATATE	23.7	14.5	4.9
8	mCT	DOTATATE	9.2	7.1	3.6					
9	mCT	DOTATATE	35.8	27	6.3					
10	mCT	DOTATATE	13.7	10.1	13.9					

ECAT = Siemens ECAT Exact 47+; mCT = Siemens Biograph mCT 64; SUV_max_ = maximum standardised uptake value; SUV_peak_ = highest SUV in a spherical region with 1 cm diameter; Volume = tumor volume measured from PET data.

PET data were utilized to estimate the tumor volumes (Table 2). The tumors were delineated with the software ROVER (ABX Germany [16]) by setting thresholds relative to the maximum SUV in the PET data sets, which provided acceptable recovery of the volumes of spheres with comparable sizes in phantom measurements (unpublished Data by MCK and HH).

Prior to PRRT, a preexisting reduced renal function was excluded using ^99m^Tc-MAG3; calculated clearance values were always >50 percent of the normal values, adjusted for age and body surface. Pregnancy was excluded by β-HCG test.

The PRRT followed a standardized protocol with infusion of 1500 mL of lysine-arginine solution (2.5% of lysine and 2.5% of arginine) starting 30 minutes at of 500 mL/hour before therapy for renal protection and standardized antiemetic medication (Ondansetron 4 mg p.o.). Radionuclide therapy was administered over 15–20 minutes using a shielded infusion pump system. Patients remained in the radionuclide therapy ward for 4–5 days.

For dosimetry, whole body scans with a [^177^Lu] standard were performed daily during hospitalization starting on the day of radionuclide administration (Siemens Ecam Duet, medium energy collimator, 20% window around the 208 keV peak, matrix 256 × 1024, scan speed 20 cm/min) and decay kinetics in the total body and the whole tumor were determined. Additionally, a SPECT/CT of the head was acquired 4–5 days after treatment (Siemens Symbia T2, equipped with a medium energy collimator). SPECT/CT data were reconstructed iteratively (3D-OSEM, 6 subsets, 6 iterations, Gauss filter of 8 mm); both attenuation and scatter were corrected for. The sensitivity of the SPECT/CT scanner in counts/second/kBq was determined from calibration with ^177^Lu activity standards in a head phantom. For technical reasons, SPECT/CT could not be performed in one patient. Assuming equal decay kinetics in all voxels of the tumor, the tumor decay function was normalized to the absolute activity value measured by SPECT/CT for the voxel with the highest uptake to deduce the maximum voxel dose D_vox_.

### External beam radiotherapy

EBRT was initiated no later than 9 days (median 2 days) after discharge of the patient from the radionuclide therapy ward.

**Figure 1:**
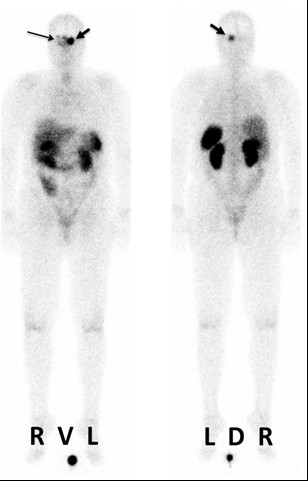
**Intensity modulated radiotherapy isodose map of patient no. 2.** This patient has a large meningioma with bitemporal spread surrounding the optic nerves and chiasm. Shown are transversal (upper left), sagittal (lower left) and coronal (right) views. In order to err on the side of caution due to the estimated 6.6 Gy PRRT dose, the optic tract was limited to 45 Gy (thick green line) and the PTV encompassing dose was limited to 49 Gy (thick orange line) instead of the originally planned 54 Gy.

SSR-PET and contrast enhanced volumetric MR-images were coregistered with the planning CT for all patients and respective GTV (gross tumor volume), CTV (clinical target volume) and PTV (planning target volume) delineated. Intensity-modulated radiation therapy plans were implemented in all cases. 1.8–2.0 Gy were delivered to the D95 surrounding the PTV to a total dose of 40–60 Gy. The total dose depended not only upon treatment volume, location and previous RT, but also on the calculated best estimate of dose from the PRRT at the discretion of the responsible radiation oncologist (RAS, MF). Thus, if vision was to be preserved, the maximum dose for the optic organs at risk was 54 Gy (D01) but, depending on previous RT or calculated PRRT-dose, also substantially lower (i.e.only 45 Gy in patient 2 as shown in Figure 1). All patients were fixated in thermoplastic masks; treatment was image guided with at least weekly conebeam CT [17].

### Evaluation of toxicity and tumor response

Evaluation preceding therapy included standard documentation of the patient’s history and physical examination. Serum chemistry and a complete blood cell count were obtained. Drug- and radiotherapy-related adverse events and toxicities were evaluated according to the Common Toxicity Criteria of the National Cancer Institute (version 4.0). At four weeks and 3–6 months after therapy, symptoms also were reassessed in the outpatient clinic of the department of radiation oncology; at these occasions serum chemistry and a complete blood cell count were obtained again. For tumor evaluation, contrast enhanced MR imaging was performed for all patients; <25% volume change was considered stable disease, >25% volume increase progressive disease and >25% decrease was deemed partial remission. SSR-PET was performed additionally after 3 – 6 months in 7 patients.

### Statistical methods

Statistical Package for Social Sciences (SPSS version 19.0; SPSS Inc.) was used for statistical analyses. Continuous variables with a normal distribution were recorded as mean ± standard deviation (SD), for those without normal distribution, the median and the range are mentioned. For the assessment of the correlation uptakes in PET and doses due to PRRT, a Spearman rank test was used. A *P*-value of 0.05 or smaller was considered to indicate significance.

## Results

### PRRT

**Figure 2:**
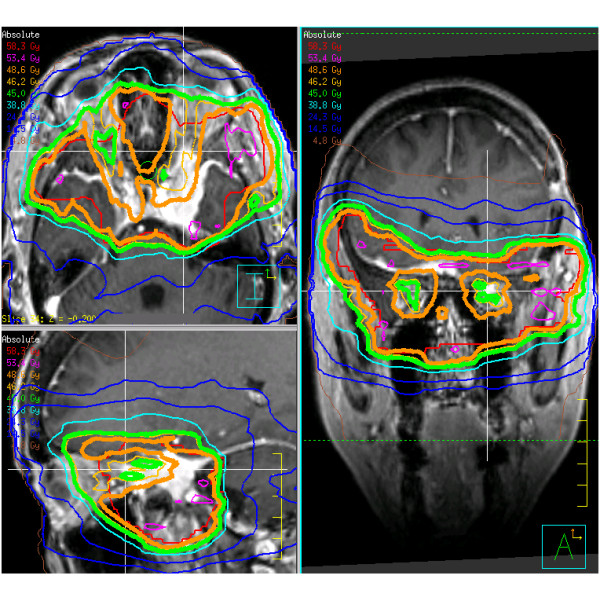
**Post-therapeutic whole body scans (anterior and posterior views) obtained 23 hours after PRRT of patient 2.** Strong persistent uptake of the radiotherapeutic agent can be observed in the meningioma (thick arrow), which also extends to the contralateral side (thin arrow).

7.4±0.3 GBq of ^177^Lu-DOTATOC/-DOTATATE were administered (Table 3). While mean total body residence time was 25.7 ± 7.7 h (range: 14.7–39.3 h), maximum activities in the tumor per 0.11 ³ voxel at 1 hour p.a. back-extrapolated from SPECT/CT ranged from 3 to 557 kBq corresponding to 0.4*10^-6^ to 70.5*10^-6^ of the administered activity. The mean lifetime of the activity in the voxels was 63.0 ± 17.7 h (range: 29.7–94.2 h). The resulting tumor dose showed a large variation (median: 7.2 Gy; range 0.2–30.6) (Table 3). A high correlation was observed between the uptakes in PET and PRRT (Spearman’s rank test: *P* > 0.01). Figure 2 illustrates the posttherapeutic scintigraphic whole body images acquired in one patient. No acute toxicities were observed during PRRT.

**Table 3:** Summary of the patients’ therapies and follow-up results

**Pat. no.**	^**177**^**Lu- DOTATOC/ DOTATATE**	**Therapeutic activity (GBq)**	**Whole body residence time (h)**	**Maximum voxel activity (kBq)#**	**Lifetime in Tumor (h)§**	**Maximum absorbed voxel dose (Gy)**	**Gy/GBq**	**EBRT target dose (Gy)**	**No. of fractions used for EBRT dose delivery**	**Change of EBRT dose due to PRRT (Gy)**	**Follow-up MR**	**Follow–up symptoms**
1	DOTATOC	7.0	39.3	71	72.4	4.0	0.57	60	30	0	No change	Improvement of pituitary insufficiency,decreased vision unchanged
2	DOTATOC	7.3	19.1	277	29.7	6.6	0.90	48.6	27	−7	No change	Retroorbital pressure fully resolved
3	DOTATOC	7.5	25.7	NA	60.1	4.0*	0.53	50	28	−4	No change	Cranial nerve palsy fully resolved
4	DOTATOC	7.2	14.7	3	94.2	0.2	0.03	54	30	0	Complete remission	Exophthalmus fully resolved
5	DOTATOC	7.6	17.1	191	44.0	6.7	0.88	54	30	0	No change	Partial loss of vision, decresed general performance constant
6	DOTATOC	7.9	22.1	539	52.4	22.3	2.82	42	22	−11	No change	Headache, hypesthesia improved
7	DOTATATE	7.9	27.2	557	68.2	30.7	3.89	60	30	0	No change	Hemiparesis unchanged
8	DOTATATE	7.2	26.6	124	74.6	7.2	1.00	40	20	0	No change	Paresis left leg unchanged
9	DOTATATE	7.3	32.3	305	69.6	16.6	2.27	54	30	0	Partial remission	Epilepsy, tremor worsened, new hearing problems, resolved to pretherapeutic state after 2 months
10	DOTATATE	7.4	32.5	161	64.8	8.2	1.11	52	20	−6	No change	Dizziness, ptosis constant

# at 1 hour p.a. in 0.11 ³ voxel volume.

§ tumor residence time normalized to initial uptake.

* no SPECT/CT data available, value deduced from PET uptake.

### EBRT

A median dose of 53.0 Gy (range 41.8–60.0) was administered in 26.7±4.3 fractions using IMRT (Table 3, Figure 1). The radiation dose delivered by PRRT showed large variation; the EBRT target dose was significantly reduced from the planned dose in four of the ten patients because of concerns about anterior optic pathway toxicity due to the summation of doses (Table 3, Figure 1).

**Figure 3:**
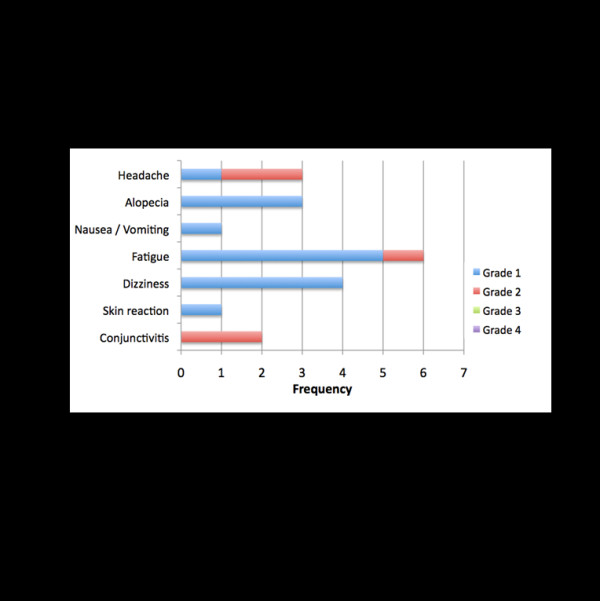
Side effects during EBRT according to CTCAE 4.0.

The other patients received EBRT as planned, irrespective of the PRRT, since the cumulative dose to neighboring organs at risk was deemed not critical by the responsible radiation oncologists. No unusual toxicities were observed during radiotherapy (Figure 3).

### Follow-up

As of November 2011 patients had a follow up of a median 13.4 months (range 4.3 months–17.0 months). All patients were alive.

One patient (no. 9) reported a temporary worsening of symptoms about 3–5 months after treatment, which could not be objectified clinically; on MRI, the tumor showed a good partial remission (>50%). Four patients reported an improvement of their symptoms. On morphological imaging, tumor size was stable in 8/10 patients; surprisingly, a complete remission was reported in patient nr.4 on the MRI acquired three months after EBRT. In the 7 patients who had a follow-up SSR-PET, PET based volume estimates decreased to 81% ± 21% (range: 46%–107%; p = 0.029) of the original size. In all of these 7 patients, the SUV values were higher after therapy as compared to the baseline PET. SUV_peak_ increased by a median of 34% (range: 8%–53%); the corresponding value for SUV_max_ was 37% (range: 15%–46%).

No chronic effects > Grade 1 have been observed to date after PRRT and EBRT; laboratory values remained unchanged during follow-up.

## Discussion

To the best of our knowledge, this is the first description of combined external beam- PRRT for meningiomas. The combination of these two therapeutic modalities has only been described once in literature; Burdick et al. combined EBRT with ^90^Y ibritumomab tiuxetan in relapsed or refractory bulky follicular lymphoma [18]. They concluded that EBRT, used to pretreat bulky sites, may improve clinical outcomes and potentially extend survival when combined with radioimmunotherapy. Furthermore, several preclinical mouse and rat model studies of heterotopic and orthotopic glioblastomas have also found a positive synergistic effect of radionuclide therapy and EBRT [19,20]: by combining both, a stronger reduction in tumor mass was measured than achieved with either treatment modality alone.

Bartolomei et al. reported on single-modality-PRRT using ^90^Y-DOTATOC as salvage monotherapy in patients with advanced meningioma. Even though therapy was well tolerated, the efficacy was limited, which most likely can be attributed to the limited dose delivered to the tumor [15]. Recently, a successful treatment of metastatic anaplastic meningioma with ^177^Lu-DOTATATE has also been reported [14].

The small number of patients and short follow up preclude a definitive answer regarding the efficacy of the combination of EBRT and PRRT treatment. The overall positive results (8 patients with morphologically stable disease, one with partial and one with complete response) after a median follow-up of 12.8 months are promising. They compare favorably to the results obtained by using the non-radioactive somatostatin analogue octreotide [21,22] and the study by Bartolomei et al. [15]. Of course, direct comparison of this study with ours is difficult due to a different patient collective and follow up. However, the short-term results in our study appear to be superior even though the achieved doses are lower than those published for PRRT in neuroendocrine tumors [23].

The current study furthermore has the limitation that we, just like other hospitals, were forced to change from the one somatostatin analogue (DOTATOC) to another one (DOTATATE) due to a change in availability of the GMP-grade precursor. Even though the difference between the two peptides is very small, it results in known alterations of receptor binding and whole body kinetics. Studies comparing the two peptides yield contradictory results on which peptide is superior for clinical use [24,25]. Nonetheless, as the different peptides do not differ in their dosimetric methodology, this will not affect the validity of the PRRT dosimetry.

**Figure 4:**
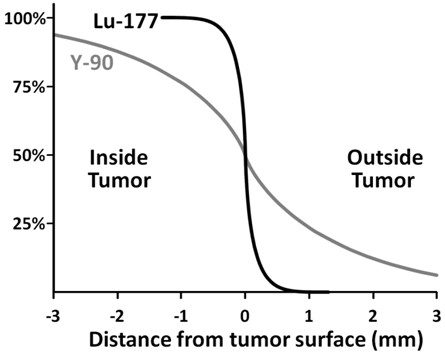
**Model dose distribution (absorbed dose) comparing peptide radionuclide therapy with**^**177**^**Lu and**^**90**^**Y respectively.** A homogeneous radionuclide dose distribution inside the tumor is assumed.

In contrast to prior larger studies we opted to use ^177^Lu as the therapeutic radionuclide even though the alternative, ^90^Y, has a six-fold higher energy deposition per decay event. This is partly compensated for by the higher activity that can be safely administered and the longer physical half-life of ^177^Lu. The use of ^177^Lu has several advantages. Firstly, due to the co-emitted gamma rays, dosimetry is easily feasible with ^177^Lu but challenging with the pure beta emitter ^90^Y, especially in smaller structures. Secondly, renal toxicity is lower for ^177^Lu [26], which is particularly important for patients with a longer life expectancy. Thirdly and most decisive, ^177^Lu has a much steeper dose gradient curve than ^90^Y. Figure 4 shows a dose distribution at the surface of a tumor with homogeneous ^177^Lu or 90Y activity uptake normalized to the maximum absorbed dose inside the tumor. The calculations are based on point dose kernels published in [27]. The relative dose decreases more rapidly with ^177^Lu than with ^90^Y. Tissue penetration outside the tumor is negligible for ^177^Lu; at a distance of 0.3 mm from the tumor surface, the dose delivered by radionuclide therapy is less than 10% of the maximal tumor dose. At least in theory, this should spare critical tissues immediately abutting the tumor (such as cranial nerves and the chiasm). The isotope and activity to be used to treat a specific patient should therefore be selected based on individual parameters such as tumor site, prognosis, radio peptide uptake, renal function, and life expectancy.

In order to avoid severe adverse effects, we chose to err on the side of caution and consequently the target EBRT dose was reduced in four of the patients with tumors very close to critical structures (Figure 1). However, only long term observations and further trials will allow better prediction of whether such a measure is truly necessary as well as which patients will attain a significant dose benefit from such a combined treatment.

Since radiotherapy as a stand-alone therapeutic modality has a high success and disease control rate even in large skull base meningiomas [28,29], a combined therapy might be most advantageous in advanced cases with tumors in proximity to radiosensitive structures such as the optic nerve, in the re-irradiation setting and for high-grade meningioma.

For a comparison of the absorbed doses of the two treatment modalities (EBBRT and PRRT), several authors suggest the application of the biologically equivalent dose concept [30-32]. In the framework of the present study, the quantities needed for the calculation of the tumor BED were unclear, so the absorbed doses of both treatment modalities were added. Future studies are needed to obtain the parameters for robust BED calculation.

An interesting finding is the increased uptake of Ga-DOTATATE/DOTATOC in meningiomas after the combined therapeutic approach. This was observed in all 7 patients who were reimaged after therapy (Table 2). In order to minimize the effects of the usage of different PET scanners we assessed both the SUV_max_ and the SUV_peak_ (the mean SUV in a spherical VOI of 1 cm diameter). The increase was observed for both parameters, so a significant effect of the equipment used appears unlikely. Haug et al. reported in a study on well differentiated neuroendocrine tumors that the tumor-to-spleen SUV ratio was an independent predictor of a longer progression free survival (PFS) after one cycle of PRRT. However, the change of absolute SUV_max_ did not correlate with PFS [33]. In our study, the tumor-to-spleen-ratio was not calculated because the spleen was not in the field of view. On the other hand, it is known from preclinical studies that neuroendocrine tumors recurring after PRRT have an increased SSR expression [33]. The observation of a SUV_max_ increase in our patients is not necessarily an indicator of increased receptor expression. Other possible explanations might be an increased blood flow induced by EBRT, the reduction in active tumor (cell) volume after therapy as observed by PET delineation or by reparative/reactive post-radiation changes. When corrected for individual tumor shrinkage, the SUV remains almost unchanged after therapy with a mean deviation of 5% ± 24% (range: -31%–32%). Since no patient progressed during follow-up, no conclusion can be drawn from the present data whether the increase in SUV correlates with prognosis as reported in neuroendocrine tumors in the paper by Haug et al. [33]. However, as a consequence from the observation of generally increasing SUV, PRRT could also be performed after EBRT; either as first cycle or additionally as a second cycle.

## Conclusions

Our experiences gained in this pilot phase indicate that peptide receptor radionuclide therapy of meningiomas may be safely used in combination with external beam radiation therapy. This approach represents an attractive strategy for the treatment of irresectable locally recurring, progressive or symptomatic meningioma in order to either increase the dose delivered to the tumor and / or to reduce the dose for organs at risk. Further studies are warranted on the basis of these results.

## Competing interests

None of the authors has a conflict of interest; no financial support was received for this project.

## Authors’ contribution

MCK: Conceived the combined approach, treated the patients with PRRT, collected and analyzed data, wrote and revised the manuscript. HH: Conceived the combined therapy approach, performed dosimetry in the context of PRRT, wrote parts of and revised manuscript, MLoe: Was involved in the treatment of the patients, revised the manuscript. FAV: Collected and analyzed data, revised the manuscript. MS: Performed the synthesis of ^177^Lu-DOTATATE/-TOC, revised the manuscript. MLa: Conceived the combined therapy approach, revised the manuscript. CR: Gave valuable input on the study conception, oversaw the treatments, and revised the manuscript. SSS: Conceived the combined therapy approach, revised the manuscript, provided the basis for somatostatin receptor PET and PRRT. AKB: Gave valuable input on the manuscript, oversaw treatments, revised the manuscript. MF: Gave input on the study conception, oversaw the treatments, performed radiotherapy, and revised the manuscript. RAS: Conceived the combined approach, performed radiotherapy, collected and analyzed data wrote and revised the manuscript. All authors read and approved the final manuscript.
